# This is not the way: global directional cues do not improve spatial learning in an immersive virtual environment

**DOI:** 10.1186/s41235-025-00654-1

**Published:** 2025-08-07

**Authors:** Ece Yüksel, Zachary Boogaart, Steven M. Weisberg

**Affiliations:** 1 Department of Psychology, University of Florida, 945 Center Dr, Gainesville, FL 32603 USA; 2 The University of Miami Miller School of Medicine, University of Miami, Coral Gables, USA

**Keywords:** Spatial learning, Global directional cues, Reference frames, Large-scale environment, Virtual reality

## Abstract

Spatial navigation relies on extracting environmental information to determine where to go. To support navigation behavior, navigational aids, such as maps, compasses, or global positioning systems (GPSs), offer access to easily extractible information, but do these aids enhance spatial memory? Here, we propose the hypothesis that navigation aids support navigation behavior when they are available but do not necessarily enhance navigation by improving the memory of a space. For example, a compass provides a global reference direction and bearing, showing where north is but may not result in a more accurate representation of an environment without the compass. We present two experiments evaluating whether people learned a large-scale, immersive virtual environment better when provided with a global reference direction. We explored whether participants used the provided reference direction to anchor their mental representation of the environment, i.e., whether their alignment of their mental map matched the cued direction. In the first (preregistered) experiment, we found no evidence of a difference in spatial memory performance between those with the compass available and those without (*n* = 54). The second experiment (*n* = 67) also revealed no difference in participants’ environmental knowledge between a compass condition or a mountain range, which provided a global directional cue in a more salient and concrete form. The exploratory results revealed that the participants did not use either cue as a reference direction. Our results inform theories on how reference directions support navigation and, more broadly, how external cues are incorporated (or not) into cognitive representations.

**Supplementary Information:**

The online version contains supplementary material available at 10.1186/s41235-025-00654-1.

To navigate effectively, a person must know where they are (location), which direction they are facing (orientation), and where they want to go (goal). Navigational aids, including tools such as compasses, maps, street signs, and global positioning systems (GPSs), offer external cues to support navigation by making one or more of these properties easily available (Weisberg et al., 2023a). Theoretically, navigational aids are one example of extended cognition—the notion that operations outside the brain may constitute important representational properties (Kiverstein, 2018). Taking a compass as an example, the navigation aid allows the navigator to keep track of the direction of the north without having to rely on internal mental resources. By offloading the computation and tracking of an abstract direction from internal processes to an external aid, the navigator can then use more resources to focus on other aspects of learning and integrating the spatial layout. Research on navigational aids, while most directly relevant to helping people navigate, can also illustrate fundamental properties for how tools support cognition in a domain with high ecological validity. From an applied point of view, the current discourse on navigational aids (including GPS) emphasizes their role in worsening spatial abilities (Dahmani & Bohbot, 2020; Hejtmánek et al., 2018; Ishikawa et al., 2008; Münzer et al., 2012; Ruginski et al., 2019). For example, long-term GPS use is associated with worse spatial transformations (Ruginski et al., 2019), while a meta-analysis showed negative associations between self-reported GPS use and environmental knowledge (Miola et al., 2024). In these experiments, navigational aids, by replacing internal computations with external cues, remove the requirement that the spatial layout is learned by the navigator at all; and thus, when they are asked to remember it later, show poorer spatial memory. Here, we report two experiments offering an important counterpoint, showing how navigational aids could be used to support spatial cognition but only when they provide access to useful information.

However, how do navigational aids support navigation? We suggest two alternatives: the access hypothesis and the construction hypothesis. Navigational aids may support spatial navigation behavior by providing access to spatial information that a person has already encoded. In contrast, navigational aids may support spatial navigation behavior by providing information that encourages people to construct more accurate or more useful spatial representations. In either case, the salience of the navigational cue and whether it is connected abstractly or concretely to the environment could affect whether it is used at all.

Different navigational aids provide different kinds of spatial information to support different types of navigation (Weisberg et al., 2023a). Here, we focus on reference directions as specified by global directional cues. Reference directions are often supported by global directional cues such as north (e.g., compasses) or distal landmarks (e.g., a mountain range or tall building). Reference directions help people establish a reference frame—an orienting device relative to which the locations and positions of landmarks and oneself can be placed. Reference frames are characteristic of how people conceptualize and represent large- and small-scale environments (Roskos-Ewoldsen et al., 1998). In contrast to other forms of spatial information, reference directions are potentially usable across multiple locations, making them theoretically important for linking segments of the environment explored separately, a process termed spatial integration (Weisberg & Newcombe, 2016). For example, a compass offers a stable north direction at every location in the environment. In contrast, a map is useful only for a region depicted on the map. Therefore, people can use this global direction cue to integrate different parts of the environment as a unified spatial layout. Global reference directions provide a consistent reference frame that navigators can use to encode the environment. This global reference frame could ease the difficulty in relating orientations of non-overlapping sub-environments to one another, thereby facilitating the integration of separate routes or areas into a single, coherent spatial layout. Supporting this idea, Meilinger and colleagues (2014) found that orientations parallel to the global reference frame resulted in better direction judgments than oblique orientations.

Reference frames constitute an important part of spatial representations and thus might be a critical way to support navigation behavior. Consistent with the notion of reference frames, spatial memories are encoded in a viewpoint-dependent manner (Albert et al., 1999; Diwadkar & McNamara, 1997; Evans & Pezdek, 1980; Meilinger et al., 2014; Mou & McNamara, 2002; Mou et al., 2004; Roskos-Ewoldsen et al., 1998; Shelton & McNamara, 2001, 2004). In one experiment, participants learned an array of objects from a single perspective and then were tested on how quickly they could recognize the array from a variety of perspectives. Their response latency was a linear function of the angular distance between the encoded view and the test view. These data suggest that people learn spatial relationships relative to a specific reference direction. However, what anchors people’s reference direction? In the case of room-size spaces, the reference direction is often (although not always) the direction the person first encounters. But reference directions are also a hallmark of large-scale spatial environments such as cities. Initially, researchers proposed that large-scale environments exhibit orientation-dependent encoding because people view them on a map rather than navigating through the environment itself (Evans & Pezdek, 1980). More recent research has shown orientation-dependent encoding, which could be relative to north (Frankenstein et al., 2012), although not always (Marchette et al., 2011).

Thus, although north is one possible reference direction, environments may differ in their preferred orientation based on ecological differences in which cues are available. In support of this notion, Weisberg and colleagues (2018) showed that providing a compass^1^ selectively improved participants’ spatial knowledge for familiar landmarks around a large city but not for recently learned landmarks around an indoor environment. One interpretation of these data is that the familiar landmarks were already encoded in a north-oriented fashion, and the compass provided access to north as a reference direction. In contrast, people did not use the compass to construct a north-oriented representation of landmarks around the indoor environment.

Here, in two experiments, we leveraged an immersive outdoor virtual environment to evaluate two types of directional cues and determine whether they improved spatial memory. First, we evaluated a compass, which provides sensory information about an abstract direction (north) and is most similar to compasses used in daily life. Second, we integrated a mountain range into the same virtual environment, which offers the same type of information—a global reference direction—in a more salient form that is embedded in the environment. Varying the type of cue allowed us to test whether the directional cue type anchored participants’ reference frames. If we observed better spatial knowledge for the compass and mountain conditions than for the baseline, this would support the notion of a general benefit for directional cues, therefore supporting the construction hypothesis. If we found better spatial knowledge for the mountain compared to the compass, this finding would support the idea that the directional cue needed to be salient, stable, and integrated with the environment rather than abstract (salience hypothesis). The construction hypothesis further predicts that if participants performed better in the directional cue conditions, reference directions would be linked to the directional cue (i.e., their map would be oriented with the directional cue at the top). Null results would provide further evidence in favor of the access hypothesis, particularly if alternate preferred reference directions could be identified that guided participants’ spatial memories, whether a directional cue was present or not.

## Experiment 1: Compass as a global directional cue

### Method

#### Participants

We recruited 65 participants (36 females; age *M* = 18.5, *SD* = 1.57) through the SONA research pool at the University of Florida. The participants received course credit in exchange for participation. Due to equipment specifications, participants needed to be between 4′8″ and 6′5″ and weigh less than 285 lbs. The participants also had normal or corrected-to-normal vision and were between 18 and 35 years old. Six participants (all females) could not finish the virtual environment learning phase, as they became motion sick and preferred to stop. Three participants did not finish the tasks due to technical issues with the equipment. Model-building data were not recorded for two participants because of technical issues. The final sample included 56 participants (31 females) for the pointing task and 54 participants (30 females) for the model-building task.

We recruited participants for a full semester for each experiment where our sample size justification was based on laboratory resource constraints. To the best of our knowledge, no suitable prior effect size was available for power estimation. Weisberg and colleagues’ (2018) used a vibrotactile compass in a somewhat related task, but the task and dependent measures were not comparable enough to justify using their effect size. In that experiment, participants pointed to external landmarks in the city, after learning the layout of an indoor environment.

#### Equipment

**Fig. 1:**
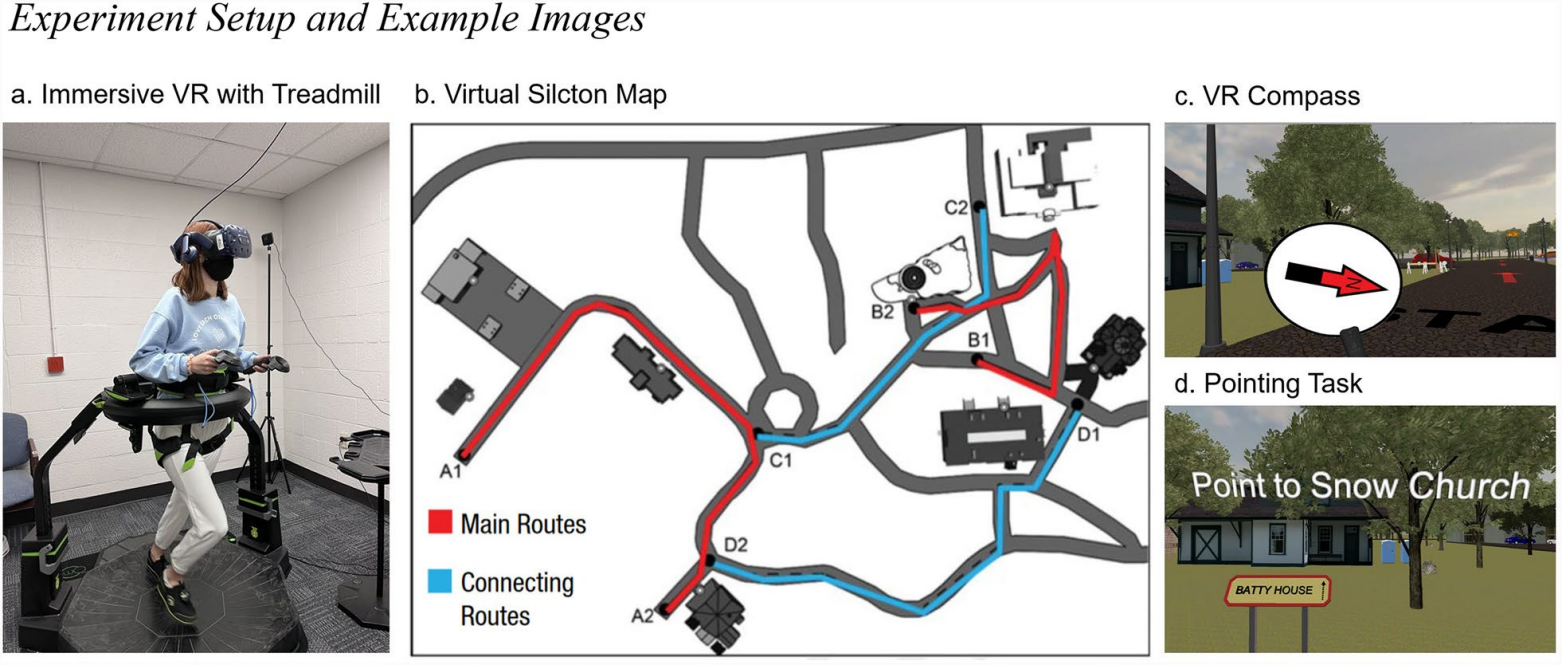
Experiment Setup and Example Images. *Note*. **a** A research assistant demonstrates the use of the head-mounted HTC Vive display and the Virtuix Omni treadmill. **b** Virtual environment (Virtual Silcton) map reproduced with permission (Weisberg et al., 2014). **c** A demo of the virtual reality compass attached to one VR controller. **d** An example of the pointing task, with a prompt indicating the building to point to

During the virtual environment portions of the experiment, participants wore an HTC Vive Pro head-mounted display, which offered 1440 × 1600 pixels per eye, a 90 Hz refresh rate, and a 110-degree field of view (HTC Corporation; https://www.vive.com/us/). Movement in the environment was controlled by a Virtuix Omni treadmill (https://www.virtuix.com/), which enabled participants to walk with full-body movements in the virtual environment. They could move freely 360 degrees in the virtual environment. The subjects were secured on the treadmill with a harness around their waist and legs, similar to a climbing harness (see Fig. 1a.). They could turn around freely within the real world and therefore walk in any direction within the virtual environment. An anti-friction-coated concave walking platform enabled the subjects to walk in place. The participants wore special shoes with inertial measurement unit tracking devices attached to Omni shoes, which allowed the participants to slide their feet to generate movement. Their steps were tracked and recreated in virtual reality. The equipment was controlled by a desktop computer with an Intel Core i9 CPU @ 3.70 GHz running Microsoft Windows 10.

*Demographics Survey*. The participants were asked to report their sex, gender, age, ethnicity, education level, whether English was their first language, and handedness.

*Santa Barbara Sense of Direction Test* (SBSOD; Hegarty et al., 2002). This self-report questionnaire consists of 15 7-point Likert scale items measuring participants’ sense of their own navigation ability. The items include ‘I am very good at giving directions’ and ‘I very easily get lost in a new city’.

*Navigation Strategies Questionnaire* (NSQ; Zhong & Kozhevnikov, 2016). This self-report questionnaire consisted of 18 multiple-choice items about participants' preferred navigation strategies, such as ‘When planning a route, do you picture a map of your route, or do you picture scenes of what you will see along the way?’ and ‘Do you picture traveling a route at the street level or from a bird's eye view?’.

*Video Game Questionnaire.* This self-report questionnaire consists of 14 items with multiple-choice and open-ended questions such as ‘Do you currently have a device to play video games?’ and ‘How often (approximately) do you currently play video games involving navigation, walking, or driving?’.

*Virtual Silcton (*Weisberg et al., 2014*).* Virtual Silcton is a 3D virtual environment replica of a real-world college campus created in Unity3D (www.unity3d.com) and is used to measure spatial navigation ability (Schinazi et al., 2013; Weisberg et al., 2014). In the current experiments, Virtual Silcton was administered via an omnidirectional treadmill with an immersive head-mounted display running SteamVR (https://store.steampowered.com/app/250820/SteamVR/, version 1.24.7). Aside from the head-mounted display and treadmill and the exceptions noted below, we used procedures similar to those used in previously published work on Virtual Silcton (Weisberg et al., 2014; Weisberg et al., 2023b; Weisberg & Newcombe, 2018).

Prior to learning Virtual Silcton, the participants were trained on using the omnidirectional treadmill and had time to adapt to the head-mounted display. In the training task, the participants had as much time as they wanted to walk around a virtual environment with grass and trees. After the experimenter was told that the participant felt comfortable walking, the experiment started.

After training, Virtual Silcton comprised two phases: a learning phase and a testing phase. In the learning phase, participants walked along predetermined paths and learned the names and locations of eight target buildings. The participants were instructed to learn the locations of the buildings as they related to each other. They took four walks: two main routes and two connecting routes. All routes were marked with red arrows on the road and were bounded by invisible walls, preventing participants from walking off the routes. The learning phase was untimed so that the participants could adequately familiarize themselves with the environment.

The target buildings were marked with an orange gem hovering above the path. In addition to the gem, a sign indicated the name of the building. Each main route included four target buildings that the participants needed to learn. The connecting routes connected the two main routes but did not have any new buildings to learn (see Fig. 1b.). The order of the two main routes and two connecting routes were randomized across participants, but the main routes always preceded the connecting routes. The participants were informed prior to the learning phase about the testing phase—that they would be performing a pointing task and a model-building task.

The participants were pseudo-randomly assigned to one of two conditions. In the compass condition, during the learning and testing phases, a compass appeared attached to the VR controller in the virtual environment (see Fig. 1c.). When the controller entered the field of view of the head-mounted display, a compass appeared, with an arrow indicating the direction of north, which coincided with the starting direction of the first route. As the compass appeared attached to the controller, the participants were in control of how frequently they would refer to it (i.e., it was not always in view). The participants were instructed that the red arrow on the compass pointed in the same direction in all scenes and that it did not refer to the actual world direction of north. The remaining participants were assigned to the control condition. The participants in the control condition were asked to hold onto one controller during the learning phase and not receive any global directional cues.

After completing the learning phase, the participants completed a testing phase consisting of two tasks—an onsite pointing task and a model-building task. In the onsite pointing task, the subjects were placed in front of the first building of Route-A. The participants could look around but could not move. A prompt at the top of the screen indicated which building they should point to (see Fig. 1d.). The participants were handed a second controller with a virtual laser extending from it. For participants in the compass condition, the other controller still showed the compass. The participants were instructed to point the laser in the direction of the front door of the named building and press the trigger button on their controller. After pointing to all the other buildings in a random order, they were automatically dropped at the next building along Route-A, followed by all the buildings from Route-B (consistent with previous Virtual Silcton testing, e.g., Weisberg et al., 2014).

After the pointing task, the subjects were instructed to construct a map of the environment. On a desktop computer, participants viewed a box that appeared on screen, which they were told represented a bird’s-eye view of the environment. The participants were instructed to drag and drop eight buildings into the box where they believed they were in the virtual world. Scrolling over each of the buildings displayed a front view of the building and its name. The participants had as long as they liked to complete both tasks.

#### Procedure

**Fig. 2:**
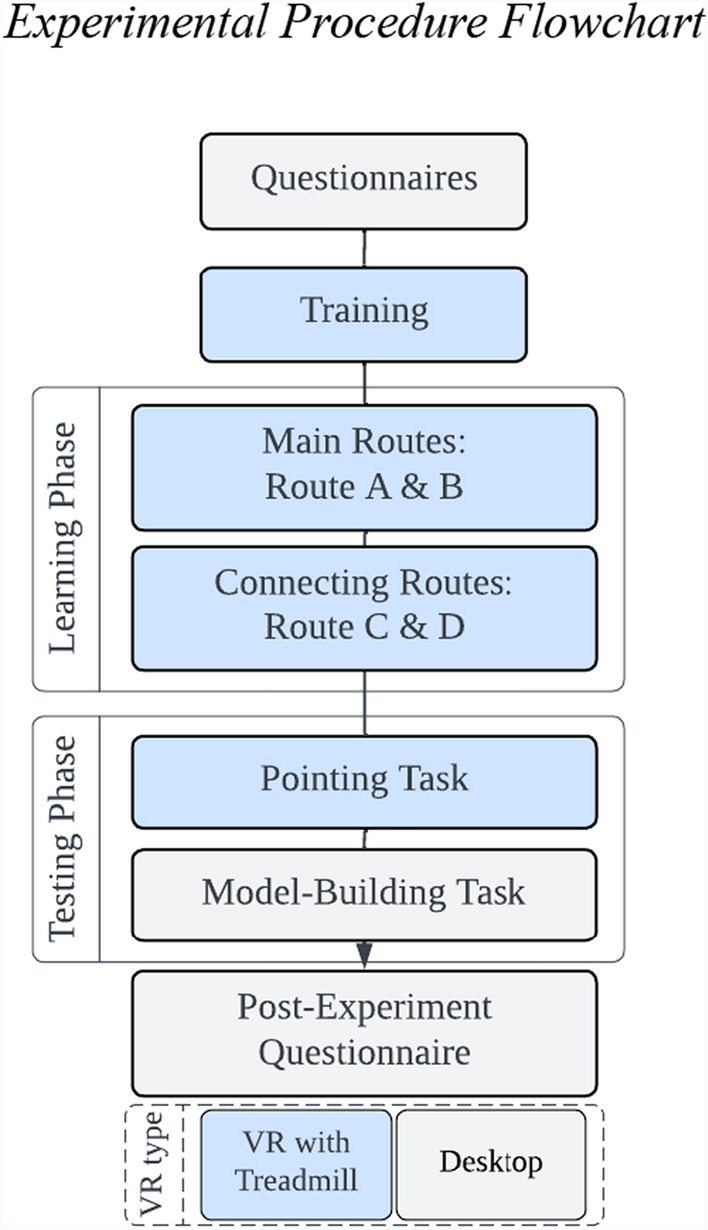
Experimental procedure flowchart. *Note.* Experimental procedure flowchart. The participants completed the questionnaires and then trained in VR. The learning phase included two main routes and two connecting routes. The testing phase consisted of pointing and model-building tasks. After the post-experiment questionnaire, the experiment was completed. The training, learning, and pointing tasks were in VR (blue rectangles), whereas the questionnaires, model-building, and post-experiment questionnaires were on desktop (white rectangles)

First, after the experimenter explained the study and the risks, the participants signed an informed consent form. Next, the participants responded to questionnaires in Qualtrics on a desktop computer (demographics, SBSOD, NSQ, video game questionnaire; see Fig. 2). After that, the participants were introduced to the immersive VR setup. The experimenter demonstrated how to wear the head-mounted display and walk on the treadmill. After the headset was calibrated on the basis of the participants’ height, the participants entered the training scene to familiarize themselves with the VR setup. The participants walked around until they verbally confirmed that they were comfortable walking. After the training, the experimenter started Virtual Silcton. Once the learning phase and pointing task were completed in VR, the participants switched to the desktop and completed the model-building task. Finally, the participants completed a post-experiment questionnaire asking about their experience and additional questions about their experience with compasses and with VR in general. The participants were then debriefed and released. The experiment lasted approximately one hour.

## Results

### Confirmatory results: Does providing a compass improve spatial learning?

As specified in our preregistration (https://aspredicted.org/blind.php?x=Z2B_X2X), our primary prediction was that having a compass available would result in more accurate representations of the environment. To test this prediction, we compared spatial knowledge between participants who had the compass available (during learning and at test) and those who did not. We assessed spatial knowledge based on performance on the pointing task and the model-building task.

For the pointing task, we calculated each participant’s average pointing error. For each pointing trial, we calculated the minimum absolute value of the angular difference between the participant’s response and that trial’s correct angle. Random guessing would result in an error of 90°. There were 56 pointing trials in total (pointing from each target building to every other target building). A one-sample t-test showed that overall, the participants learned the locations of the buildings significantly better than chance, *t*(55) = -21.58, *p* < 0.001, *d* = 2.88. However, we observed broad variability in the pointing task (*M* = 39.88, *SD* = 17.38).

**Fig. 3:**
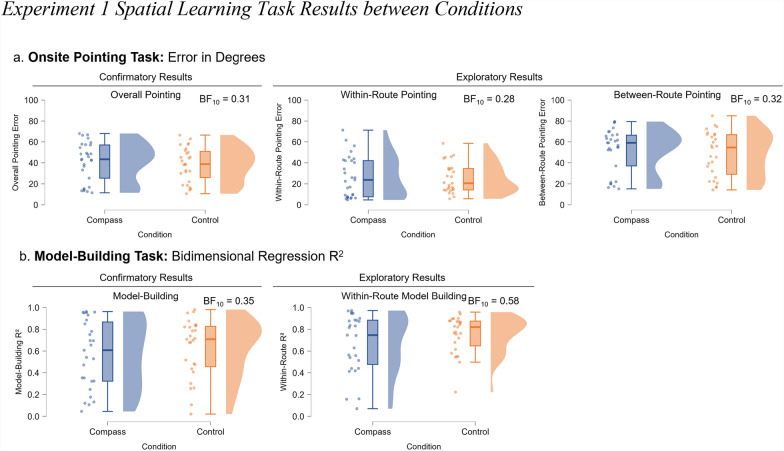
Experiment 1 spatial learning task results between conditions. *Note.* The results for Experiment 1 comparing the compass (dark blue) and control (orange) conditions. (a) From left to right, raincloud plots display the results (angular error) for overall pointing, within-route pointing, and between-route pointing. (b) From left to right, raincloud plots display the results (bidimensional regression) for the configuration of the participants’ overall map and their within-route map

To test our primary hypothesis that providing a compass would result in more accurate spatial knowledge, we performed a Bayesian independent samples t-test for the overall pointing error with the compass condition as a between-subjects factor (compass group *n* = 29; control group *n* = 27). We used a default Cauchy prior on the effect size centered at 0 with a scale of 0.707, as implemented in JASP (version 0.19.3). There was moderate evidence in favor of the null hypothesis (no difference between the compass and control conditions; BF_10_ = 0.31; Fig. 3a.). This means the data were approximately three times more likely under the null hypothesis than under the alternative hypothesis. The participants with access to a compass (*M* = 41.14, *SD* = 18.50) did not point more accurately than those without the compass (*M* = 38.54, *SD* = 16.34). According to common interpretive guidelines (Van Doorn et al., 2021), a Bayes Factor between 0.33 and 0.10 can be considered moderate evidence for the null, and this result falls at the upper end of that range. We apply the same interpretive scale consistently throughout the manuscript to qualify both evidence in favor of the null and alternative hypotheses. We also include the prior and posterior, Bayes factor robustness check, and sequential analysis graphs in all JASP files for analyses.

For the model-building task, we applied the bidimensional regression (Friedman & Kohler, 2003; Tobler, 2010) formula to assess model accuracy. Bidimensional regression computes the correlation between two sets of coordinates—the correct locations of buildings and the participants’ placements of buildings—controlling for differences in scale, rotation, and translation between the two sets. In other words, bidimensional regression provides a measure of the configurational accuracy of a map (e.g., a map could be rotated in any direction with no change in error). The correlation between the adjusted points and the correct response yields a correlation coefficient, R^2^, which is the proportion of variance explained in the actual layout of buildings by the participants’ maps.

As with the pointing task, we conducted a Bayesian independent samples t-test for the model-building R^2^ between conditions (compass group *n* = 27; control group *n* = 27). Again, there was moderate evidence in favor of the null hypothesis (no difference between the compass and control conditions; BF_10_ = 0.35; Fig. 3b). The accuracy in the compass condition (*M* = 0.57, *SD* = 0.31) was no better than that in the control condition (*M* = 0.63, *SD* = 0.27).

Based on the preregistered analyses, the *construction* hypothesis was not supported; we did not observe improved spatial memory when the participants were provided with a compass in a novel environment.

### Exploratory results: within and between-route

One possibility is that adding a reference direction improved spatial integration. Virtual Silcton offers the opportunity to investigate multiple types of spatial knowledge, including how people learn locations of buildings that are traveled directly (within-route) versus buildings whose spatial relation must be inferred (between-route). We tested whether there was an effect of the compass for pointing error separately for within-route and between-route pointing. Within-route pointing error is the mean of pointing error to buildings along one of the first two main routes. The between-route pointing error is the pointing error from buildings along one main route to buildings along the other main route. Again, we found anecdotal evidence for the null hypothesis for within-route pointing error (BF_10_ = 0.28; compass group *n* = 29, *M* = 26.62, *SD* = 19.52; control group *n* = 27, *M* = 25.20, *SD* = 14.13; see Fig. 3a.) and moderate evidence for the null between-route pointing error (BF_10_ = 0.32; compass group *M* = 52.02, *SD* = 21.00; control group *M* = 48.54, *SD* = 21.38).

Similarly, we computed the within-route accuracy (R^2^) on the model-building task.^2^ To do so, we took the mean of each bidimensional regression score calculated separately for the set of four buildings along the initial two main routes. Bayesian independent t-tests revealed anecdotal evidence for the null hypothesis (BF_10_ = 0.58; compass group *M* = 0.67, *SD* = 0.27; control group *M* = 0.75, *SD* = 0.17). These exploratory results are consistent with the confirmatory results that the compass did not promote better learning even when it was available during the test.

## Experiment 1 discussion

Overall, the participants did not construct more accurate maps with a compass available in a large-scale virtual environment. These findings provide evidence against the construction hypothesis. Our hypothesis for providing participants with a global directional cue in Virtual Silcton was that the cue would help subjects to integrate the landmarks within and between routes. Since our experiment investigates large-scale navigation, most of the landmarks are not intervisible. Considering this aspect of the environment and the tasks, providing participants with an integration device (the global direction cue) for helping the participant construct a unified representation, should have been useful. A global directional cue, in principle, could allow people to encode the environment in reference to a clear orienting direction, which they could also view during the pointing task; however, they did not use that reference direction to construct a more accurate spatial representation. These results are consistent with research on providing compasses in large-scale indoor spaces (Weisberg et al., 2018). In that study, participants used the compass to access an established reference frame (a north-oriented representation of the city of Philadelphia; supporting the access hypothesis). However, they did not use the compass to encode the newly learned indoor landmarks. In both compass studies, however, the global directional cue was provided in an abstract and transient way, not linked or central to the immediate environment. In Experiment 2, we integrated another type of global directional cue into the environment, a mountain range, to evaluate whether making the cue more salient and positioned in the environment itself would lead people to use it more effectively.

## Experiment 2: mountain range as a global directional cue

Experiment 1 demonstrated that participants did not spontaneously use a compass to improve their spatial knowledge of Virtual Silcton. One possible explanation for this result is that a compass provides a global directional cue that is abstract and transient and therefore not easily accessible, as people are learning a new environment. In Experiment 2, we evaluated a corollary of the access hypothesis, the *salience* hypothesis, which posits that a reference direction will be useful only if it provides a highly salient and informative direction. For Experiment 2, we integrated a highly visible and directional mountain range into the environment to provide participants with a salient and visual directional cue. Specifically, we hypothesized that a visible and concrete (rather than abstract) directional cue would support the construction of a reference frame anchored to that cue. We refer to this as the salience hypothesis. Because we observed no difference between the compass and control conditions in Experiment 1, we recruited a new sample of participants who were randomly assigned to either the mountain condition or a compass condition in which the compass pointed in the same direction as the mountain range (although no mountain range appeared.) We elected not to include a no-cue control in this experiment because A) findings from Experiment 1 suggested that having a compass did not affect error, so it could serve as a baseline; B) including the compass condition allowed us to further investigate how people did (or did not) use the reference cues, the analyses of which are presented in a combined analysis section after Experiment 2; C) limited resources would not allow us to run more than approximately 60 participants. Finally, we also wanted to be able to measure whether participants knew where the cued directions were and which direction participants were using to orient their representations of the space during the test; thus, we did not include the cue during the test (either the compass or the mountain range) and instead asked participants to point in the direction of the cue.

## Method

### Participants

We recruited 77 (39 male, age *M* = 19.07, *SD* = 1.23) participants via the SONA research pool at the University of Florida for course credit. We recruited the same sample size as we preregistered in Experiment 1 and followed the same confirmatory preregistered analyses as our first experiment. The inclusion criteria were the same as those for Experiment 1. In Experiment 2, four participants (three females) could not complete the learning phase because of motion sickness. Six participants (two females) did not finish the tasks due to technical issues with the equipment. Model-building data were not recorded for six participants (three females) because of technical issues. The final sample included 67 participants (33 females) for the pointing task and 61 participants (30 females) for the model-building task.

#### Equipment

All equipment was the same as in Experiment 1.

#### Materials

All measures were the same other than parts of the post-experiment questionnaire, which are explained in the next section. Materials are available on the Open Science Framework (https://osf.io/wuaz3/?view_only=cc49564ec89c461483c770563732d682).

#### Procedure

The procedure was identical to Experiment 1 except as indicated above. The experiment lasted approximately one hour.

#### Virtual silcton

**Fig. 4:**
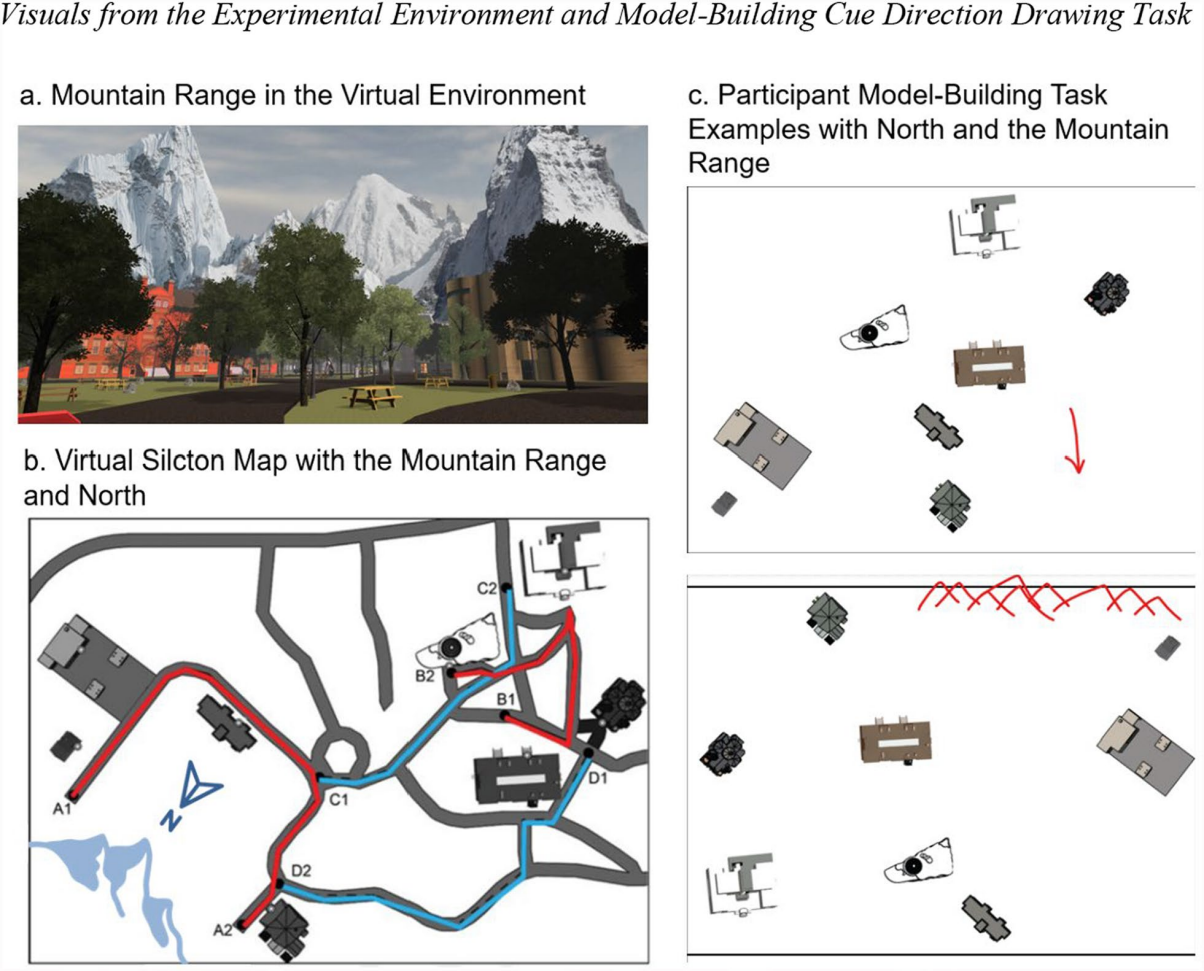
Visuals from the experimental environment and model-building cue direction drawing task. Note. (a) The mountain range added to Virtual Silcton. (b) Virtual Silcton map with the mountain range direction (light blue mountain icon) and north (dark blue north icon). (c) Examples of the participants’ model and their drawings of the cued global direction (in red). On the top is a compass group participant where north is indicated by the arrow. On the bottom is a mountain range group participant where the mountains are drawn as triangles indicating the direction of the mountain range

For the mountain range condition, the location of the mountain range was chosen such that it would be clearly visible from most of the environment (Fig. 4a.). The mountains were placed far enough from the navigable portion of Virtual Silcton that they did not provide local directional information. That is, regardless of where the participant stood in the Virtual Silcton environment, the individual features of the mountain range appeared in the same global direction. The compass condition was the same as in Experiment 1 except that the compass pointed to the middle of where the mountain range would appear. The instructions and procedures (training environment, learning phase, testing phase with onsite pointing and model-building) were the same as those in Experiment 1, with two exceptions. First, the compass and mountain range were not present during the onsite pointing task. This was done in part because providing it did not change the results in Experiment 1 so that participants could be asked to estimate where the reference direction was by pointing either to the middle of the mountain range or in the direction of the compass. Third, after the model-building task, we asked the participants to draw where they thought the compass indicated north or the direction of the mountain range on their maps (see Fig. 4c.).

#### Post-experiment questionnaire

The post-experiment questionnaire included questions about participants’ prior experience in compass use and whether and how they used the compass or the mountain range (depending on their condition) to learn about the environmental layout.

## Results

### Confirmatory results: Does a salient visual directional cue improve spatial learning?

We used the same analysis methods for the pointing and model-building tasks as in Experiment 1, with the exception that judgments toward the compass or mountain range directions were analyzed separately. In the pointing task overall, the participants learned the locations of the buildings significantly better than chance, *t*(66) = 23.40, *p* < 0.001, *d* = 2.86. However, we observed broad variability in the pointing task (*M* = 36.23, *SD* = 18.81).

**Fig. 5:**
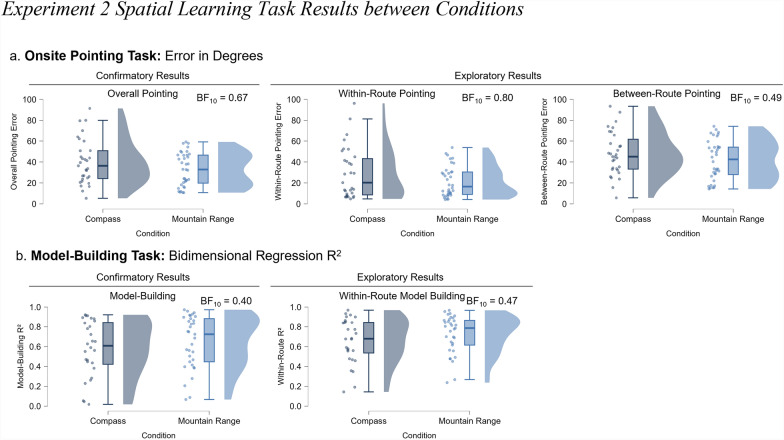
Experiment 2 spatial learning task results between conditions. Note. Results for Experiment 2, comparing the compass (dark blue) and mountain range (light blue) conditions. (a) From left to right, raincloud plots display the results (angular error) for overall pointing, within-route pointing, and between-route pointing. (b) From left to right, raincloud plots display the results (bidimensional regression) for the configuration of the participants’ overall map and their within-route map

To investigate the salience hypothesis, we compared the mountain condition with the compass condition. The salience hypothesis posits that global directional cues anchor participants’ reference frames when they are salient (operationalized here as visually apparent and situated within the environment) and that this strong anchor helps participants encode the environment better than the absence of such a cue. We therefore predicted that participants in the mountain condition would learn the environment better than participants in the compass condition (which Experiment 1 showed was not very helpful for learning). To test this prediction, we used a Bayesian independent samples t-test to analyze overall pointing error. We found only anecdotal evidence in support of the null hypothesis (BF_10_ = 0.67; Fig. 5a). In other words, the data are approximately 1.5 times more likely under the null hypothesis than under the alternative. The participants in the mountain range group (*n* = 36, *M* = 33.02, *SD* = 15.74; Fig. 5a.) did not point more accurately than participants in the compass group (*n* = 31, *M* = 39.96, *SD* = 21.51). Similarly, we found anecdotal evidence supporting the null hypothesis in model-building R^2^ (BF_10_ = 0.40; Fig. 5b.). The participants in the mountain range group (*n* = 33, *M* = 0.65, *SD* = 0.27) did not build more accurate models than did the participants in the compass group (*n* = 28, *M* = 0.58, *SD* = 0.29). While these results do not constitute strong evidence, it suggests a slight trend toward no group difference.

Providing a salient directional cue—a mountain range—in the environment did not improve spatial knowledge.

### Exploratory results

#### Within and between-route

As in Experiment 1, we assessed within- and between-route pointing to investigate whether the cue type affected the main routes and/or the overall spatial layout integration. We observed anecdotal evidence in favor of the null hypothesis for within-route pointing error (BF_10_ = 0.80; compass group *M* = 29.40, *SD* = 24.48; mountain range group *M* = 21.39, *SD* = 14.54) and between-route pointing error (BF_10_ = 0.49; compass group *M* = 47.88, *SD* = 21.04; mountain range group *M* = 41.73, *SD* = 18.96). This suggests a slight trend toward no difference between the compass and mountain range conditions, especially in between-pointing error while evidence is weaker for within-route pointing error.

For the model-building task, we also found anecdotal evidence for the null hypothesis for within-route R^2^ (BF_10_ = 0.47; compass group *M* = 0.66, *SD* = 0.23; mountain range group *M* = 0.72, *SD* = 0.19). Consistent with the confirmatory results, these analyses support the null hypothesis that a mountain range did not improve spatial knowledge.

#### Did participants encode the directional cues?

**Fig. 6:**
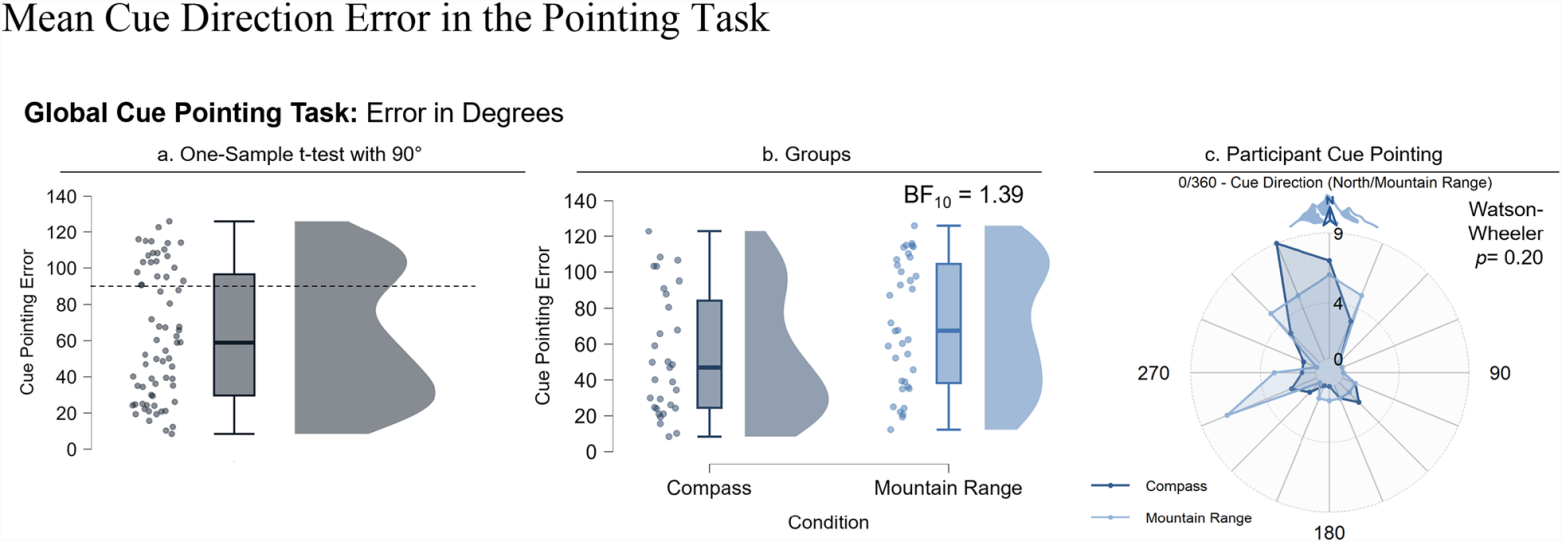
Mean cue direction error in the pointing task. Note. (a) One-sample t-test with 90°. (b) Global cue pointing error in the compass and mountain range groups. (c) Mean cue direction pointing error. 0° is the correct angle of the global directional cue direction

If participants did not use the directional cue to improve their representation of the environment, we wanted to evaluate whether they encoded the direction of the cue at all. We asked the participants to point in the direction of the cue (either the center of the mountain range or the direction indicated by the compass) at each location during the pointing task. We calculated the average angular error across each global direction pointing trial within the participant. A one-sample t-test revealed that participants pointed in the cue direction more accurately than chance (90°; *t*(66) = 6.29, *p* < 0.001, *d* = 0.77), but we observed broad variability (*M* = 62.63, *SD* = 35.63). Comparing the conditions, we found anecdotal evidence (BF_10 =_ 1.39; Fig. 6b) that the compass group was more accurate (*M* = 53.37, *SD* = 34.12, *n* = 31) than the mountain range group was (*M* = 70.61, *SD* = 35.42, *n* = 36).

**Fig. 7:**
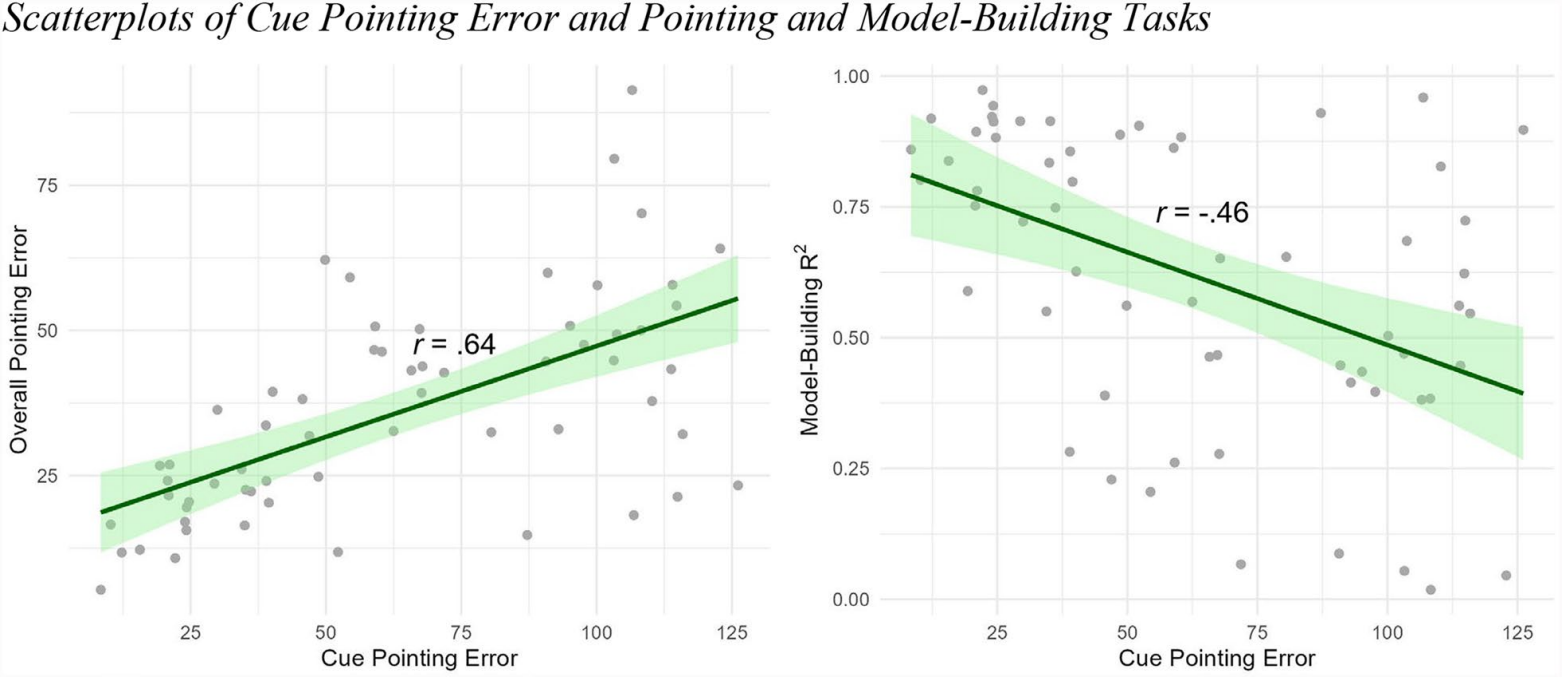
Scatterplots of cue pointing error and pointing and model-building tasks. Note. Scatterplots for cue pointing error and overall pointing error (left) and model-building R^2^ (right)

Based on the individual variability we observed in how accurately participants pointed to the directions indicated by the cues across conditions, we evaluated whether people who were able to point in the cue direction were also better at learning the environment. We found a significant correlation between cue pointing error and overall pointing error (*r*(59) = 0.64, p < 0.001) and between cue pointing error and model-building R^2^ (*r*(59) = -0.46, p < 0.001). Both correlations show that people who accurately encoded the cue direction also had more accurate configurational knowledge of Virtual Silcton (Fig. 7).

## Experiment 2 discussion

Overall, we did not find support for the salience hypothesis. A salient global directional cue (a mountain range) was not more useful than a compass (which, based on data from Experiment 1, was not more useful than no additional cue) for encoding a large-scale virtual environment. Despite not improving spatial representations of the environment overall, we also found that participants who learned the environment better overall also reported the cue direction more accurately. It could be that better navigators use the additional information to encode the environment more accurately than they would without the cue (i.e., the rich get richer) or that better navigators incidentally encode the cued direction but that it has no effect on their representations otherwise.

In addition to individual differences, we wanted to examine whether global directional cues influenced spatial reference frames. That is, did the availability of a global directional cue change the way the environment was learned to be primarily oriented to the directional cue rather than oriented to some other aspect of the environment? To address this question, we conducted a series of exploratory analyses across both Experiments 1 and 2. We had three main questions. First, did combining data across experiments (i.e., increasing statistical power) yield any differences across conditions that were not observed due to a lack of statistical power? Second, was there evidence that participants aligned their spatial representations with the global directional cue (i.e., did they use the compass or the mountains as the anchor for their reference frame)? Third, did individual variability across subjects contribute to who used global directional cues or not?

## Combined results: exploratory analyses

### Did providing global directional cues improve spatial memory?

To determine whether there was any evidence with increased sample size for the construction hypothesis, we combined data from Experiments 1 and 2. Note, this is an imperfect comparison, since we did not run a new no-cue condition in Experiment 2. However, since we recruited from the same undergraduate pool (albeit in different semesters), we believe the data are comparable. We also did not control specific aspects of the cues across studies, including where the compass pointed. If any effect of global cues generally emerged, these should be evident in combining conditions to improve statistical power. Still, these are important limitations, which is why we emphasize that these are exploratory analyses.

**Fig. 8:**
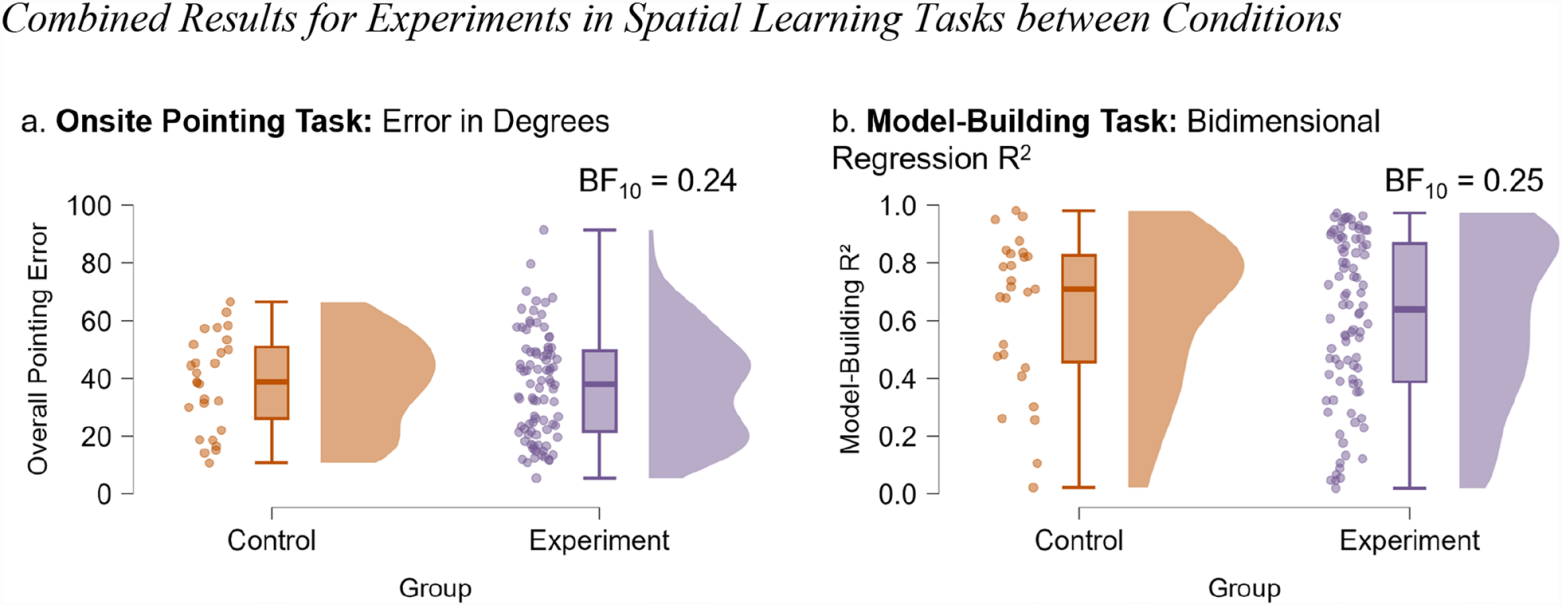
Combined results for experiments in spatial learning tasks between conditions. Note. Combined results for both experiments, comparing the control (orange) and experimental (compass and mountain range; purple) groups. (a) Raincloud plots displaying the results (angular error) for the overall pointing error. (b) Raincloud plots displaying the results (bidimensional regression) for the configuration of the participants’ overall map

We ran a Bayesian independent t-test to see whether participants in either experimental cue condition (compass or mountain range combined) outperformed participants in the control condition. For the pointing task, we found moderate evidence for the null hypothesis (BF_10_ = 0.24, Fig. 8a). The experiment group (*n* = 88, *M* = 37.33, *SD* = 18.46) did not perform differently than the control group did (*n* = 27, *M* = 38.54, *SD* = 16.34). Similarly, for the model-building R^2^, we found moderate evidence for the null hypothesis (BF_10_ = 0.25, Fig. 8b). The experiment group (M = 0.60, SD = 0.29) did not perform differently than the control group did (*n* = 27, *M* = 0.63, *SD* = 0.27).

## Reference frames: Did participants use the cued direction while learning the environment?

If the virtual compass did not improve accuracy, one possibility building off prior research is that the compass could help anchor participants’ spatial reference frames (Weisberg et al., 2018). In other words, if participants used direction cues to learn the environment, we would expect the orientation of their representations to align with that direction, even if that alignment did not result in improved memory for the configuration of the buildings. Thus, we computed the optimal rotation angle for each map (used to rotate participant maps to align with a template map via bidimensional regression calculation) to assess whether the participant’s map aligned with the directional cue. We assumed that if a participant placed the direction indicated by the cue at the top of their map, they were using it to orient their reference frame (e.g., north = up). We computed this angle for the overall map and separately for the buildings within each main route (Route-A and B; see supplemental Table S1 for circular descriptives). Figure 8 displays circular histograms or spider plots, showing the distribution of participants’ model-building angles across conditions. That is, if the cues affected the alignment of the participants’ maps, we expected to see different trends in the map alignments across the different conditions. To quantify these differences, we used circular statistics in JASP (Bahde & Berens, 2019; Agostinelli & Lund, 2017) and R to determine whether participants built different reference frames across conditions.

First, to understand whether the condition contributed to a preferred direction in Experiments 1 and 2, we ran Watson-Wheeler non-parametric tests (see supplementary Table S1 for descriptive statistics). For Experiment 1, the condition did not influence the circular mean of orientations for the participants’ map overall (W = 0.15, *p* = 0.93; Fig. 9.1a) or separately for the orientation of the Route-A landmarks (W = 0.78, *p* = 0.68; Fig. 9.1b) or Route-B landmarks (W = 0.14, *p* = 0.93; Fig. 9.1c). Similarly, for Experiment 2, the condition did not influence the circular mean for the overall orientation (W = 4.12, *p* = 0.13; Fig. 9.2a) or orientation for the Route-B landmarks (W = 1.69, *p* = 0.43; Fig. 9.2c). However, we found that for Route-A, the mountain range group and the compass group had different alignments of the route (W = 6.52, *p* < 0.05; Fig. 9.2b). For the combined results, we found no evidence for a condition effect for overall angle (W = 2.08, *p* = 0.72), Route-A (W = 5.03, *p* = 0.28), or Route-B (W = 2.80, *p* = 0.59).

**Fig. 9:**
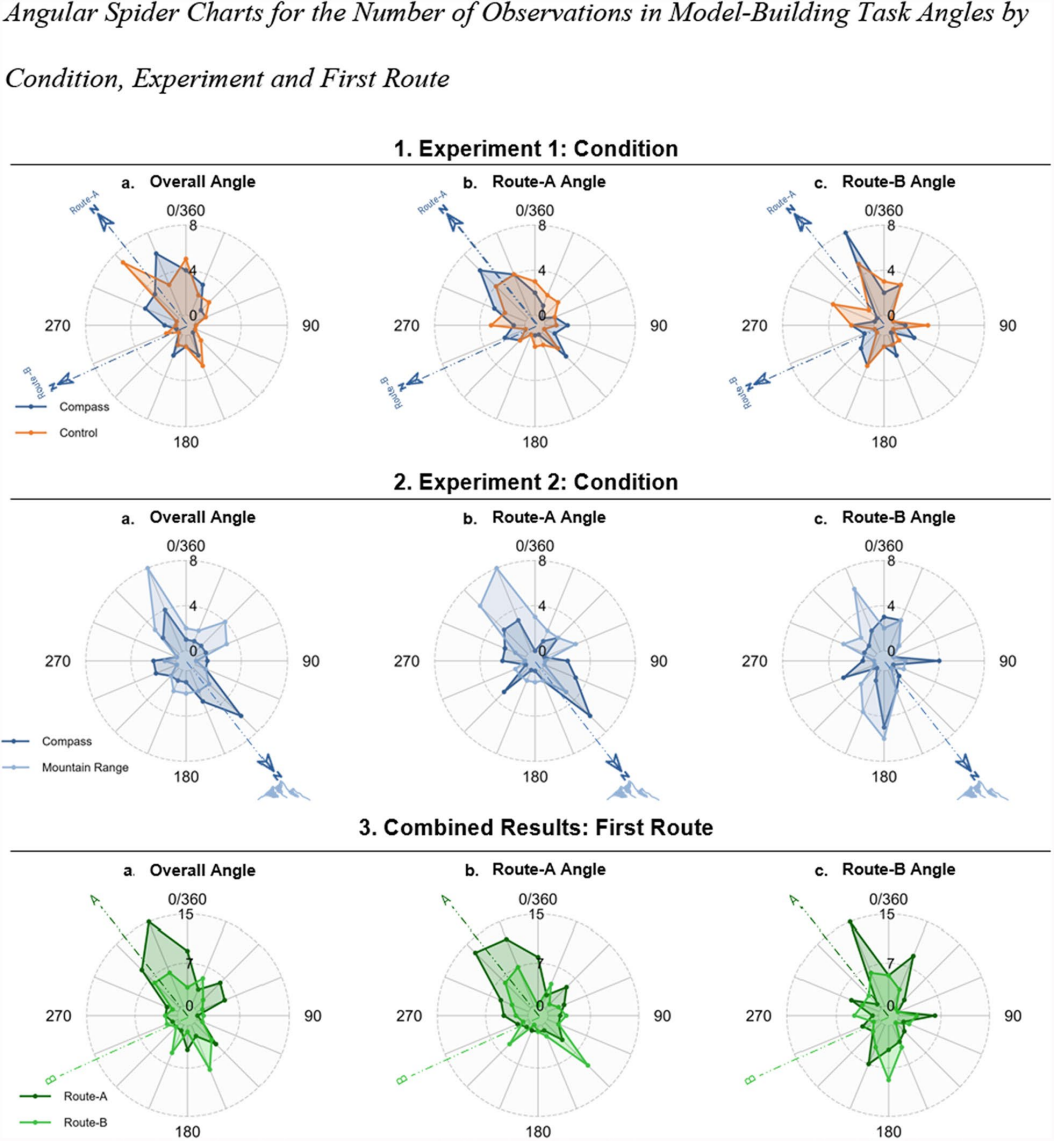
Angular spider charts for the number of observations in model-building task angles by condition, experiment and first route. Note. Number of participants’ model-building alignment angle observations in spider charts by condition and first route. The lines on the charts indicate angles ranging from 0 to 360 degrees. The minimum, middle, and maximum numbers of observations are 0, 4, and 8, for Experiment 1 and 2 and 0, 7, and 15, respectively, for the combined results, as shown in the circles. From top to bottom, Experiment 1: Conditions: compass (dark blue) and control (orange) groups. Experiment 2: compass (dark blue) and mountain range (orange) groups. Combined Results: Route-A (dark green) and Route-B (light green) groups. From left to right: (a) overall angles, (b) Route-A angle, (b) Route-B angles for all rows

Since the participants did not use the reference direction provided by the cue to orient their reference frames, we considered another possible source of reference frame selection, which is the first direction encountered in an environment. In our experiment, the participants started learning the environment either from Route-A or B. We repeated the analyses above but included the first route as a between-subjects variable to determine whether the first route had an effect on the participants’ environment reference frames. Watson–Wheeler tests revealed that the first route had an effect on the overall angle (W = 8.09, *p* < 0.05; Fig. 9.3a) and Route-A angle (W = 6.63, *p* < 0.05; Fig. 9.3b) but not on Route-B angle (W = 3.35, *p* = 0.19; Fig. 9.3c). These analyses revealed that the starting route influenced the alignment of the overall angle and Route-A angle. Figure 9.3 shows that the Route-A first route group (in dark green) used the Route-A route start angle to set up reference frames for the overall angle and Route-A.

### Individual differences

People vary widely in navigation ability (Hegarty et al., 2002; Ishikawa & Montello, 2006; Weisberg & Newcombe, 2016; Weisberg et al., 2014). Thus, one possibility we considered was that individual difference factors would contribute to directional cue use. We assessed three potential sources of individual variability: 1) sex differences; 2) differences in video game play; and 3) differences in cue use. We report SBSOD scores with spatial memory tasks in Table S5 in the supplementary material.

#### Sex differences

**Fig. 10:**
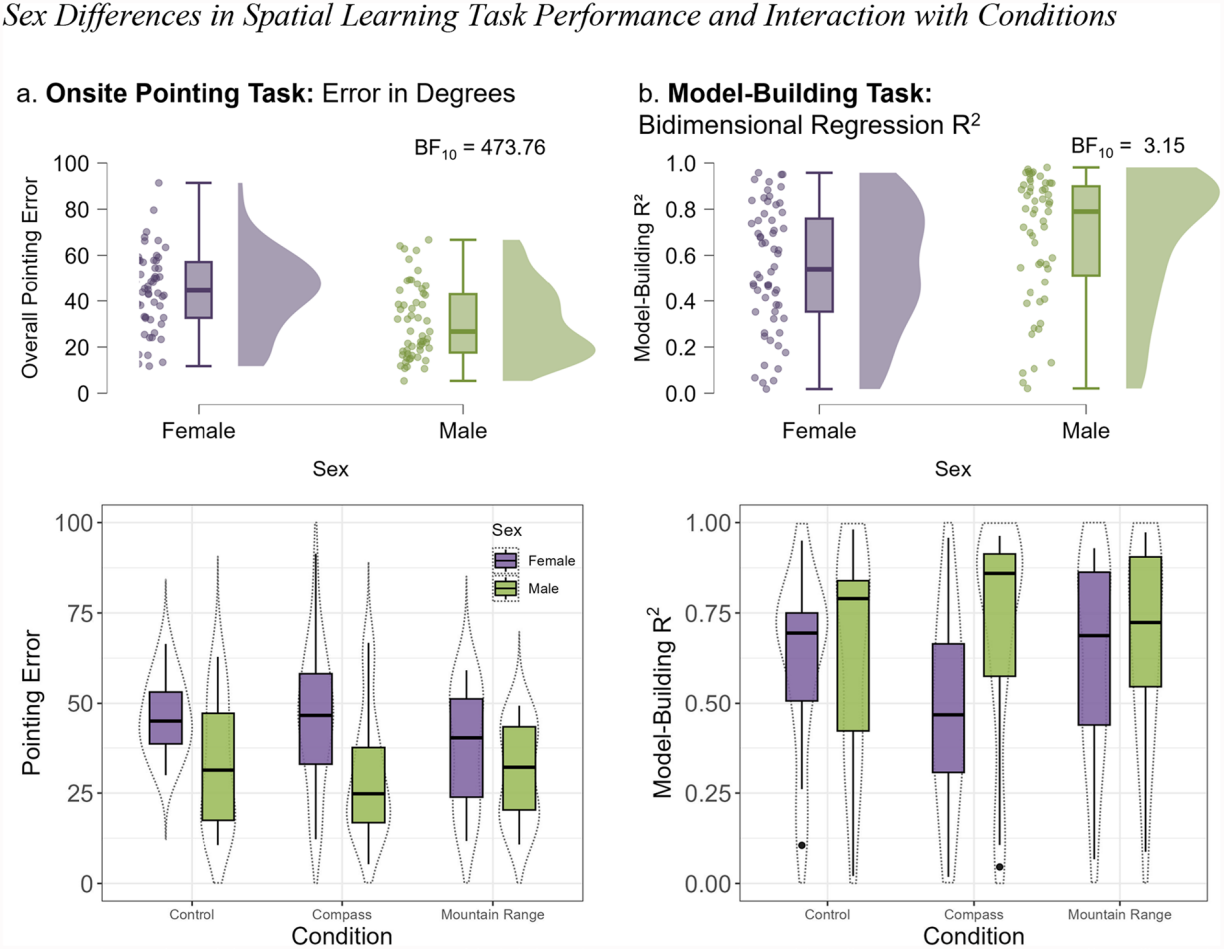
Sex differences in spatial learning task performance and interaction with conditions. Note. Combined results for both experiments, comparing the female (purple) and male (green) groups. (a) Raincloud plots displaying the results (angular error) for the overall pointing error. (b) Raincloud plots displaying the results (bidimensional regression) for the configuration of the participants’ overall map

We computed Bayesian independent samples t-tests to examine sex differences in pointing error and model-building performance. Consistent with previous research, we found that men outperformed women in both tasks. With respect to onsite points (*n* = 123, 64 females), with BF_10_ = 473.76, we found strong evidence in favor of a sex difference. In the model-building R^2^ (*n* = 115, 60 females), we observed moderate evidence in favor of a sex difference, with a BF_10_ = 3.14 (Fig. 10). This sex difference was not modulated by the condition for the pointing task (BF_incl_ = 0.32) or the model-building task (BF_incl_ = 0.23). Men outperformed women whether a directional cue was available or not.

#### Video game experience

To analyze whether video game play correlated with spatial learning performance, we computed a Bayesian independent samples t-test between video-game players and non-players. We grouped participants based on their response to a question on the video game questionnaire asking whether they currently play video games. In the pointing task, we found strong evidence in favor of the alternative hypothesis of a difference between the groups (BF_10_ = 86.14, Figure S2 in the supplementary material). Video game players performed better (*n* = 42, *M* = 29.80, *SD* = 16.48) than non-players (*n* = 73, *M* = 42.11, *SD* = 17.25). For model-building, the same pattern emerged with strong evidence for the alternative hypothesis, with a BF_10_ = 159.00. Again, video game players (*n* = 42, *M* = 0.73, *SD* = 0.23) performed better than the non-player group (*n* = 73, *M* = 0.53, *SD* = 0.28). Video game players outperformed non-players regardless of whether a directional cue was available (Video Game Play * Condition interaction had a BF_incl_ = 0.15 for pointing, BF_incl_ = 0.14 for model-building; Figure S3 in the supplementary material).

#### Compass experience

**Fig. 11:**
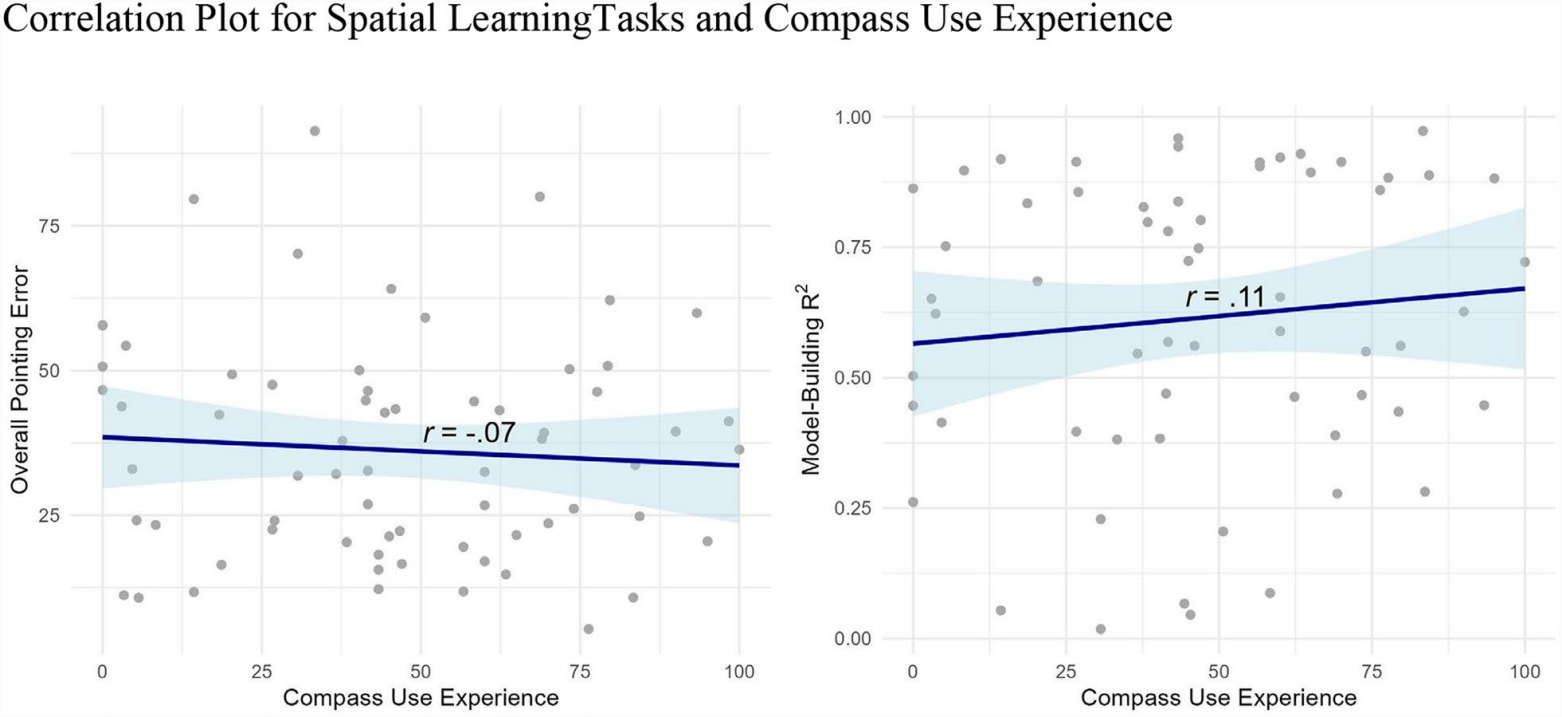
Correlation plot for spatial learningtasks and compass use experience. Note. Correlation plots for compass use experience and overall pointing error (left) and model-building R^2^ (right)

We also evaluated prior experience with compasses specifically. In the Experiment 2 post-experiment questionnaire, the participants were asked to rate their compass experience. Our first three questions were highly intercorrelated (1- I know how to use a compass, 2- I am experienced in using a compass, 3- I find it easy to use a compass to find my way), whereas our fourth question (4- In my daily life, I use the north as my reference point.) was not (see supplementary Table S4 for correlations). Therefore, we calculated the mean of the three questions to run a correlation between compass use experience and spatial learning performance. As shown in the scatter plots (Fig. 11), self-reported compass experience was not correlated with spatial learning performance. We did not observe a significant correlation between compass use and onsite pointing error (*r*(65) = -0.07, *p* = 0.55) or model-building (*r*(59) = 0.11, *p* = 0.42).

To summarize, although men outperformed women and video game players outperformed non-players, neither of these individual difference factors interacted with the cue condition, meaning that certain groups did not benefit from global direction cues more than others did. Similarly, previous experience with compass use, which ranged quite widely across participants, did not predict performance in Experiment 2, in which both conditions featured a global cue.

## General discussion

In two experiments, we evaluated whether and how global directional cues support spatial learning in a large-scale immersive virtual environment. Our aim was to examine whether providing global directional cues improved spatial memory and whether more salient cues supported spatial learning more. Our results support the notion that global directional cues do not enhance spatial memory; an effect partially attributable to the fact that people did not construct representations of the environment relative to the cued directions. Our results support the *access* hypothesis, that providing global directional cues will promote spatial learning when they allow people to access representations from a previously encoded reference direction, rather than the *construction* hypothesis that global directional cues will promote the creation of representations aligned with that direction. That is, our results undermine the construction hypothesis (participants did *not* spontaneously use these cues to build better representations) more so than they support the access hypothesis. We also did not find any evidence for the salience hypothesis, providing mountain range in the environment did not improve spatial learning performance more than a compass.

## Reference frame selection and directional cues

Confirmatory analyses, specified by our preregistration, revealed that there is no advantage to having global directional cues. In both experiments, when the participants were provided with global directional cues, their pointing and model-building task performance did not improve even combined across the experiments. Our results are consistent with those of Weisberg and colleagues (2018), who reported that participants did not use north to learn landmarks in an indoor environment but did support pointing to well-known external landmarks in the surrounding city. Access to a compass reduced pointing error to the external landmarks because participants could use their internally consistent and previously encoded city reference frame while pointing. Our results are consistent with their findings for support of the access hypothesis, suggesting that the compass did not help the participants construct better configurations, although it might have been useful for accessing spatial relationships if they were previously encoded in relation to north.

Previous research has shown that the orientation-dependent encoding of large-scale environments could be relative to north (Frankenstein et al., 2012), although not always (Marchette et al., 2011). Frankenstein and colleagues (2012) reported that participants’ pointing performance was most accurate when they were facing north in their highly familiar city environment and that deviation from a north-facing orientation resulted in an increase in pointing error. The participants relied on a north-oriented global reference frame, even though they spent significantly more time navigating the city rather than looking at maps. In contrast, Marchette and colleagues (2011) reported that their participants also clearly preferred a single reference orientation, which generally aligned with the campus’s salient building and path axes. One possible interpretation of our findings is that the participants used the structure of the environment, particularly the path from the starting point of Route-A, to encode the environment rather than north-oriented or mountain range-oriented reference frames.

What do people use to anchor their reference frames? One source of north-based bias could be our tendency to look at maps of the spaces we live in and travel to. For example, Meilinger and colleagues (2015) manipulated whether people learned an environment through a map or through virtual navigation. In that study, learning through a map led people to use a north reference frame, whereas learning the environment through navigation first showed a navigation-based reference frame, even if participants were provided with a map later. People also tend to generally associate north with up, tending to prefer south- relative to north-going routes (Brunyé et al., 2010, 2012). The hometown of the navigator and their culture might also affect a person’s selection of reference frames and navigation performance. For example, Barhorst-Cates and colleagues (2021) reported that Padua, Italy, residents performed better in pointing tasks than did residents living in Salt Lake City, Utah, despite the dominant mountain range and cardinal organization in Utah, offering a global directional cue. Furthermore, Werner and Schmidt (1999) reported that people were more accurate and much faster in identifying landmarks when they were asked to imagine themselves aligned with a major street in Gottingen than when misaligned. In our experiment, we recruited participants who attended a public university in Florida, which has no mountainous regions, although Gainesville, Florida, has a grid-like street system and is oriented with respect to the north. Still, it could be the case that the participants in our experiments did not use the mountain range because they grew up in a place where this would not have been possible.

In contrast to encoding an environment with respect to a global directional cue, people can also encode orientation according to their body-centered coordinates or experience. For example, Shelton and McNamara (2004) found that the initial heading had a strong emphasis on reference frames. People perform better in judgment of relative direction tests when their heading direction is parallel to the study view than novel views (Shelton & McNamara, 2001). In our experiments, people built their model-building maps aligned with their starting route, especially for Route-A. People who learned Route-A first aligned their overall maps in the model-building task with the initial direction they faced in the environment. This finding is consistent with previous studies showing that egocentric orientation influences environmental encoding and retrieval (Roskos-Ewoldsen et al., 1998; Shelton & McNamara, 2004). However, we did not observe this behavior in people who started encoding the environment from Route-B. This discrepancy could be due to the shape of the routes. As shown in Fig. 4b, Route-A is linear for a longer part of the route until you make your first turn compared with Route-B. This long sightline could explain why participants starting from point A1 encoded the environment in reference to the heading direction of Route-A. In Route-B, participants make the first turn at a shorter distance.

Our speculations here are similar to what Yerramsetti and colleagues (2013) found, that people may use the perceptual and experiential qualities of space while selecting headings. The authors tested participants’ pointing judgments without specifying a heading direction in a campus environment to infer the approximate heading participants assumed at each location. They reported that the assumed heading was similar across participants and seemed to depend on episodic or visual properties of the space. Similarly, Gagnon and colleagues (2014) showed that when participants start learning the environment in a north-oriented fashion, they exhibit faster learning and greater memory flexibility. The authors concluded that the alignment of world-centered and body-centered reference frames is beneficial for learning a new spatial layout. Overall, reference frame selection can depend on what the environment offers and the navigator’s strategy. Future research should investigate the relationship between the environment and the usage of global directional cues.

## Supporting versus enhancing spatial cognition

Providing participants with a global directional cue did not enhance their spatial memory. Although navigation aids could in theory provide additional information, that information is only likely to be used if participants know how to integrate it with the representations they are building on their own, using cues already available to them. Whether a particular navigational aid is useful might depend on what information the environment already provides the navigator. For example, in a dense forest, the environment itself does not provide landmarks or a global direction cue unless you are trained to look at the shape of a tree to find south or infer any other directional cue from the environment. In contrast, if the environment offers sufficient heading and orientation information, you may choose to use that information to create your representation rather than using the navigation aid providing a global directional cue such as a compass.

In our experiment, one reason why the participants did not use or need directional cues might be that the environment and landmarks provided enough information to encode the spatial layout. Similarly, path integration, the ability to keep track of one’s position and orientation via bodily self-motion information (Etienne & Jeffery, 2004), could be sufficient enough on its own or in combination with the landmark-guidance system in which visual landmarks and environmental information are used for homing, reorientation, and wayfinding (Collett, 2010; Trullier et al., 1997). Zhao and Warren (2015) showed that people integrate cues from path integration and landmark-guidance systems to reduce navigation variability. If one or both systems fail to provide enough information to the navigator, the dependency on navigational aids might be observed.

Spatial cues that encourage integration might provide better support. For instance, Brunec and colleagues (2023) reported that, in the same environment used here (Virtual Silcton), participants who explored areas with high global environmental connectivity formed more accurate cognitive maps. Similarly, learning spatial knowledge from multiple views could help participants recall information more flexibly. When participants learn an array of objects at two different angles, they encode the spatial layout relative to the view angle with two reference frames but not a single viewpoint-independent representation (Shelton & McNamara, 1997).

In short, in our experiment, participants might have had enough information from walking on the VR treadmill with body-based cues, optic flow, and landmarks within the environment to represent distance and direction. Thus, it is difficult to rule out the possibility that participants found the global direction provided ancillary to their core representation of space. The fact that participants could accurately report the cued direction showed that they observed the direction; however, this direction did not overcome the existing strong cues in the environment. Future research should reduce the number of available cues in the environment or consider how existing information might be better highlighted; for example, supporting spatial integration might be a better way to support spatial learning.

## Individual differences

Overall, we observed that men outperformed women in both the pointing and model-building tasks. This result is consistent with prior studies showing a sex difference in spatial learning tasks (Galea & Kimura, 1993; Ishikawa & Montello, 2006; Nazareth et al., 2019). However, we did not find any evidence of an interaction of sex differences in terms of cue use. We also found that playing video games is positively correlated with spatial learning performance. Video game players were able to point to buildings with less error and build more accurate maps than non-players. This finding is also consistent with previous findings (Murias et al., 2016; Ventura et al., 2013). Similar to sex differences, video game play did not interact with cue use.

Critically, we found that self-reported compass experience did not correlate with either pointing tasks or model-building tasks. This finding is important because it rules out the possibility that people did not use the compass because they did not know how. One remaining possibility that will be the subject of future investigations is whether the tasks we used required the use of global directional cues to perform optimally. Designing tasks and environments where compass use is required for success may reveal more nuanced ways in which these cues are incorporated into otherwise independent spatial representations.

## Limitations

Our experiments had several limitations. Our participants were limited in terms of age and demographic range, due to testing young, relatively healthy college students and were likely to have lived a large part of their lives in a relatively flat part of the U.S.

In terms of experimental design, the placement of the mountain range was chosen to be maximally visible within the environment, but the trade-off was that the mountain range was not particularly visible from the starting locations along each route. We also did not systematically show the environment from all orientations at the start or ensure that the participants rotated around. It is thus possible that the participants did not notice the mountain range until later. Future research should examine the effect of selecting the cue direction or trying to balance the exposure and visibility of the environmental global cue direction for every participant.

## Conclusion

Overall, we found that providing participants with global directional cues did not help them construct more accurate spatial layouts even though they were able to point in the cued direction. Further analyses revealed that participants’ spatial memory may not have improved because they did not encode the environment relative to the cue direction. Our findings highlight the importance of understanding the limitations and use of global directional cues and navigational aids.

## Supplementary Information

Additional file 1.

## Data Availability

Experiment 1 was preregistered on Aspredicted.org (https://aspredicted.org/blind.php?x=Z2B_X2X). Data were analyzed using R (R Core Team, 2021) and JASP (https://jasp-stats.org/).

## Abbreviations

VR: Virtual Reality
SBSOD: Santa Barbara Sense of Direction Scale
NSQ: Navigation Strategies Questionnaire

## Footnotes

1 This experiment used a vibrotactile compass, consisting of small motors in an array around the navigator’s arm. The motor closest to North would vibrate, providing the navigator with tactile information about the direction of North.

2 Between-route accuracy for model-building is captured by overall model-building performance reported in the confirmatory results.

## Code availability

All data, analysis code, and research materials are available on the Open Science Framework (https://doi.org/10.17605/OSF.IO/WUAZ3). The version of Virtual Silcton used in these experiments is available on Figshare (https://doi.org/10.6084/m9.figshare.25674912).
