# Supplementary material for: This is not the way: global directional cues do not improve spatial learning in an immersive virtual environment
Source: Cogn Res Princ Implic. 2025 Aug 7;10:48. doi: 10.1186/s41235-025-00654-1 (PMC12331561; doi:10.1186/s41235-025-00654-1)
Supplement: Supplementary file 1 — Additional file 1. [file 41235_2025_654_MOESM1_ESM.docx]

| **Table S1** |  |  |  |  |  |  |
| --- | --- | --- | --- | --- | --- | --- |
| *Circular Descriptives for Model-Building Task Angles* | | | | |  |  |
|  | Overall Angle | | Route-A Angle | | Route-B Angle | |
| Experiment 1 | | | | | | |
|  | Compass | Control | Compass | Control | Compass | Control |
| Mean Direction | 338.78 | 346.22 | 328.98 | 335.41 | 319.43 | 330.97 |
| Mean Resultant Length | 0.50 | 0.39 | 0.39 | 0.39 | 0.23 | 0.31 |
| Median | 333.20 | 359.08 | 327.01 | 331.15 | 342.52 | 344.60 |
| Standard Deviation | 1.18 | 1.37 | 1.38 | 1.37 | 1.72 | 1.54 |
| Experiment 2 | | | | | | |
|  | Compass | Mountains | Compass | Mountains | Compass | Mountains |
| Mean Direction | 151.46 | 12.20 | 90.05 | 348.24 | 98.66 | 269.72 |
| Mean Resultant Length | 0.12 | 0.27 | 0.08 | 0.41 | 0.03 | 0.16 |
| Median | 128.71 | 347.18 | 108.14 | 339.77 | 93.53 | 300.32 |
| Standard Deviation | 2.07 | 1.61 | 2.27 | 1.34 | 2.68 | 1.93 |
| *Note.*  If not stated otherwise, all values are calculated on a normalized period of 2π. | | | | | | |
| ᵃ Value is shown with respect to the original period. | | | |  |  |  |

| **Table S2** |  |  |  |  |  |  |
| --- | --- | --- | --- | --- | --- | --- |
| *Combined First-Route Circular Descriptives for Model-Building Task Angles* | | | | | | |
|  | Overall Angle | | Route-A Angle | | Route-B Angle | |
| First Route | Route-A | Route-B | Route-A | Route-B | Route-A | Route-B |
| Valid | 67 | 48 | 67 | 48 | 67 | 48 |
| Mean Directionᵃ | 357.1 | 302.64 | 341.3 | 348.22 | 338.8 | 256.02 |
| Mean Resultant Length | 0.41 | 0.06 | 0.45 | 0.06 | 0.22 | 0.15 |
| Medianᵃ | 343.5 | 305.91 | 335.6 | 335.83 | 342.7 | 257.93 |
| Standard Deviation | 1.34 | 2.35 | 1.26 | 2.37 | 1.75 | 1.93 |
| *Note.*  If not stated otherwise, all values are calculated on a normalized period of 2π. | | | | | | |
| ᵃ Value is shown with respect to the original period. | | | | | | |

## First route: does it affect navigation performance?

We ran a Bayesian independent t-test to see whether the first route influenced navigation performance. We only observed anecdotal evidence for the alternative hypothesis in Route-A R^2^ based on first route (see Table S3). Participants who started learning the environment starting from Route-A, performed better in the model-building task’s Route-A modeling (BF_10_ = 1.79).

**Table S3**

| ***Descriptive Statistics for First Route Groups*** | | | | | | | | | | | | | | | | | | | | |  |
| --- | --- | --- | --- | --- | --- | --- | --- | --- | --- | --- | --- | --- | --- | --- | --- | --- | --- | --- | --- | --- | --- |
|  | | | | | | | | | | | | | | | | | **95% Credible Interval** | | | |  |
|  | | **Group** | **N** | | **Mean** | | **SD** | | | **SE** | | | **Coefficient of variation** | | | | **Lower** | | **Upper** | |  |
| Pointing Error |  | Route-A |  | 71 |  | 36.579 | |  | 18.064 | |  | 2.144 | |  | 0.494 |  | | 32.303 |  | 40.854 |  |
|  |  | Route-B |  | 52 |  | 39.689 | |  | 18.390 | |  | 2.550 | |  | 0.463 |  | | 34.569 |  | 44.808 |  |
| Within Pointing |  | Route-A |  | 71 |  | 23.952 | |  | 18.356 | |  | 2.178 | |  | 0.766 |  | | 19.607 |  | 28.297 |  |
|  |  | Route-B |  | 52 |  | 27.568 | |  | 18.983 | |  | 2.632 | |  | 0.689 |  | | 22.283 |  | 32.852 |  |
| Between Pointing |  | Route-A |  | 71 |  | 46.049 | |  | 20.596 | |  | 2.444 | |  | 0.447 |  | | 41.174 |  | 50.924 |  |
|  |  | Route-B |  | 52 |  | 48.780 | |  | 20.757 | |  | 2.879 | |  | 0.426 |  | | 43.001 |  | 54.558 |  |
| Model-Building R^2^ |  | Route-A |  | 67 |  | 0.632 | |  | 0.283 | |  | 0.035 | |  | 0.448 |  | | 0.563 |  | 0.701 |  |
|  |  | Route-B |  | 48 |  | 0.572 | |  | 0.276 | |  | 0.040 | |  | 0.482 |  | | 0.492 |  | 0.652 |  |
| Route-A R^2^ |  | Route-A |  | 67 |  | 0.745 | |  | 0.260 | |  | 0.032 | |  | 0.348 |  | | 0.682 |  | 0.808 |  |
|  |  | Route-B |  | 48 |  | 0.630 | |  | 0.295 | |  | 0.043 | |  | 0.468 |  | | 0.544 |  | 0.715 |  |
| Route-B R^2^ |  | Route-A |  | 67 |  | 0.702 | |  | 0.278 | |  | 0.034 | |  | 0.396 |  | | 0.634 |  | 0.770 |  |
|  |  | Route-B |  | 48 |  | 0.713 | |  | 0.281 | |  | 0.041 | |  | 0.394 |  | | 0.632 |  | 0.795 |  |
|  | | | | | | | | | | | | | | | | | | | | |  |

**Pointing to the Cue Direction**

We calculated the circular mean for each participant’s cue direction across all trials (that is, each time they pointed to the global direction) and plotted them with the correct North/Mountain Range direction as 0 angle for consistency (Figure 6c.). For example, for a person who consistently pointed exactly to the correct North direction, their cue direction circular mean would be 0. We see from the plot that there were participants who were able to point in the right cue direction. The Compass group’s mean pointing direction was 340.41° with a SD of 1.10, whereas the Mountain Range group had a mean pointing direction of 314.91° and SD of 1.22. To assess if the circular mean pointing direction was different between groups, we ran a Watson-Wheeler test. The results showed that there is not a significant difference between the groups (W(2) = 3.24, p = 0.20).

**Figure S1**

*Combined Results for Experiments in Spatial Navigation Tasks and Video Game Play Experience*


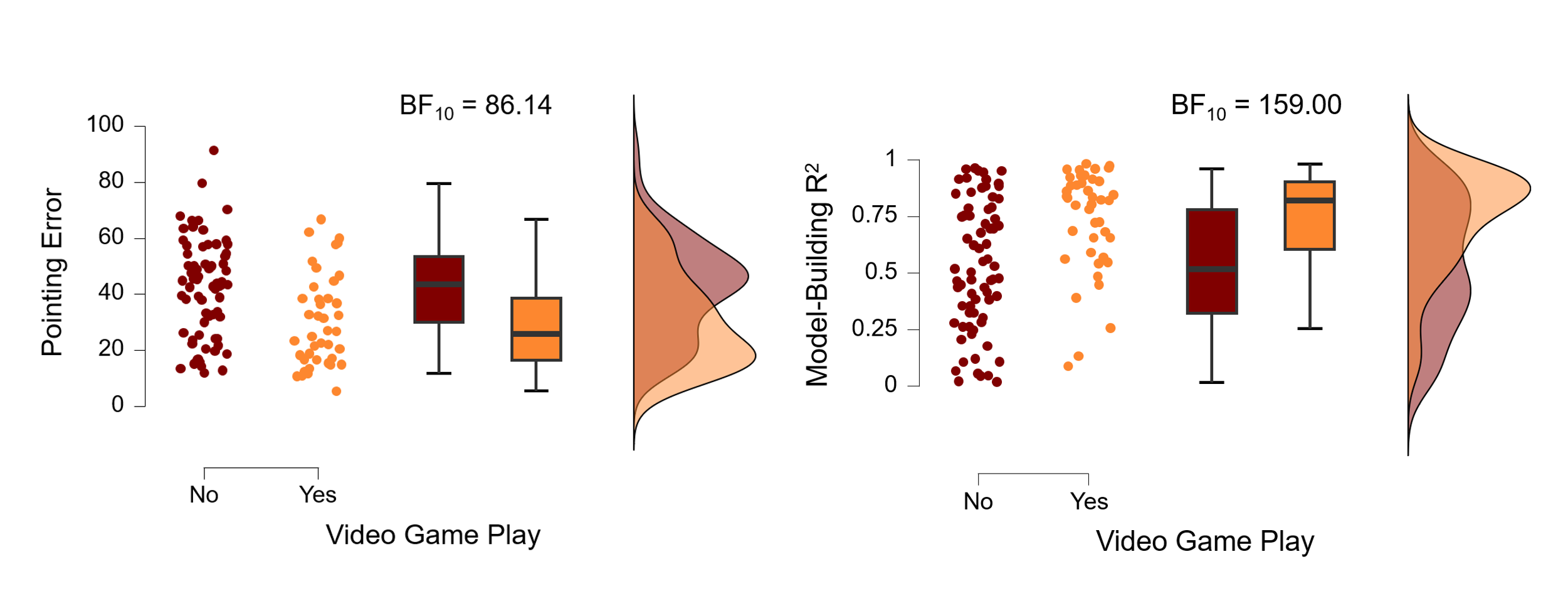


*Note*. Combined results for both experiments, comparing navigation task performance by video game play.

**Figure S2**

*Combined Results for Experiments in Spatial Navigation Tasks and Video Game Play Interaction*


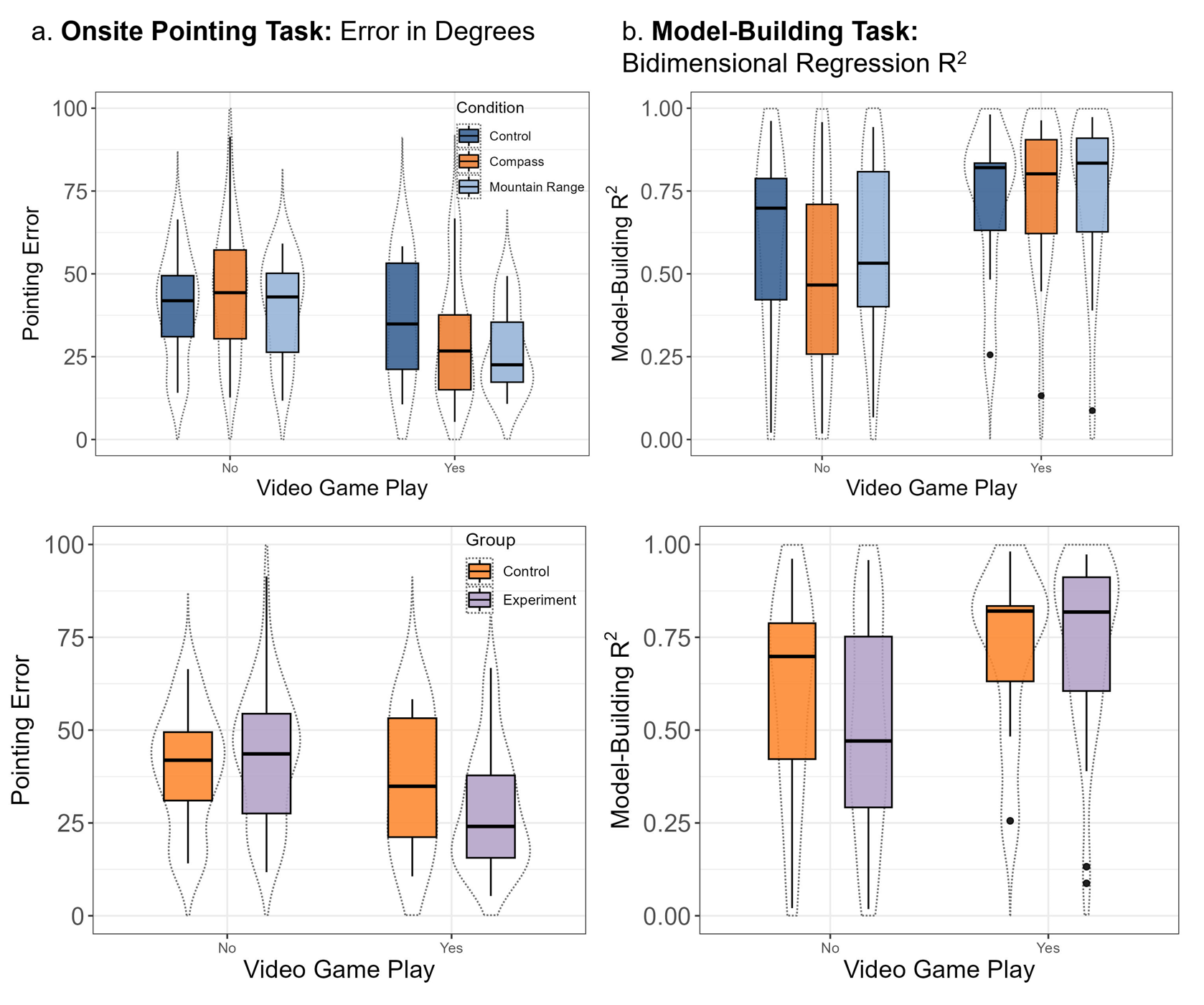


*Note*. Combined results for both experiments, comparing navigation task performance in conditions (first row); Control or Experiment groups (second row). (a) Raincloud plots display results (angular error) for overall pointing error. (b) Raincloud plots display results (bidimensional regression) for the configuration of participants’ overall map.

| **Table S4**  *Compass Use Questionnaire Questions Correlations* | | | | |  |
| --- | --- | --- | --- | --- | --- |
|  | know_how_use | experienced | easy_use | use_north_daily |  |
| know_how_use | 1.00 | 0.69 | 0.69 | 0.17 |  |
| experienced | 0.69 | 1.00 | 0.74 | 0.25 |  |
| easy_use | 0.69 | 0.74 | 1.00 | 0.35 |  |
| use_north_daily | 0.17 | 0.25 | 0.35 | 1.00 |  |

**SBSOD: Correlation Between Sense of Direction and Navigation**

We ran correlations between navigation performance tasks and SBSOD to see if self-report wayfinding abilities correlated with the participants’ performance and within the navigation tasks. In SBSOD, participants’ score can range from 1 (poor sense of direction) to 7 (good sense of direction). Therefore, we expected to see better navigation performance in higher SBOSD score participants. Overall, we observed that navigation tasks are highly correlated and self-report wayfinding behavior in SBSOD also correlates with navigation performance. We report the full correlation matrix in Table S5.

| **Table S5** |  |  |  | |  | |  | |  | |  | | | | |  | | |  | | | |  | | |  |  |  | |
| --- | --- | --- | --- | --- | --- | --- | --- | --- | --- | --- | --- | --- | --- | --- | --- | --- | --- | --- | --- | --- | --- | --- | --- | --- | --- | --- | --- | --- | --- |
| *SBSOD Correlations with Spatial Task Performance* | | | | | | | | | | | | | | | | | | | | | | | | | | | | |  |
| Variable | Pointing  Error | Within Pointing | | Between Pointing | | Model-Building R^2^ | | Route- A R^2^ | | |  | Route-B R^2^ | |  | SBSOD | | | | | | |  |  |  |  |  |  |  |  |
| 1. Pointing Error | — |  | |  | |  | |  | | |  | | | | | | |  | | |  | | |  |  |  |  |  |  |
| 2. Within Pointing | 0.88*** | — | |  | |  | |  | | |  | | | | | | |  | | |  | | |  |  |  |  |  |  |
| 3. Between Pointing | 0.95*** | 0.68*** | | — | |  | |  | | |  | | | | | | |  | | |  | | |  |  |  |  |  |  |
| 4. Overall R^2^ | -0.76 | -0.63*** | | -0.74*** | | — | |  | | |  | | | | | | |  | | |  | | |  |  |  |  |  |  |
| 5. Route-A R^2^ | -0.58 | -0.52*** | | -0.55*** | | 0.6*** | | — | | |  | | | | | | |  | | |  | | |  |  |  |  |  |  |
| 6. Route-B R^2^ | 9.36×10^-3^ | -0.03 | | 0.04 | | -0.07 | | -0.26** | | | — | | | | | | |  | | |  | | |  |  |  |  |  |  |
| 7. SBSOD | -0.27** | -0.27** | | -0.23* | | 0.11 | | 0.13 | |  | | | 0.03 | | | |  | | | — | | | | |  |  |  |  |  |
| * p < .05, ** p < .01, *** p < .001 | | | | | | | | | | | | | | | | | | | | | | | | | | | | |  |
